# Genetics and Gene Expression Involving Stress and Distress Pathways in Fibromyalgia with and without Comorbid Chronic Fatigue Syndrome

**DOI:** 10.1155/2012/427869

**Published:** 2011-09-29

**Authors:** Kathleen C. Light, Andrea T. White, Scott Tadler, Eli Iacob, Alan R. Light

**Affiliations:** Departments of Anesthesiology, Neurobiology and Anatomy, and Exercise and Sport Science, The University of Utah, Salt Lake City, UT 84132, USA

## Abstract

In complex multisymptom disorders like fibromyalgia syndrome (FMS) and chronic fatigue syndrome (CFS) that are defined primarily by subjective symptoms, genetic and gene expression profiles can provide very useful objective information. This paper summarizes research on genes that may be linked to increased susceptibility in developing and maintaining these disorders, and research on resting and stressor-evoked changes in leukocyte gene expression, highlighting physiological pathways linked to stress and distress. These include the adrenergic nervous system, the hypothalamic-pituitary-adrenal axis and serotonergic pathways, and exercise responsive metabolite-detecting ion channels. The findings to date provide some support for both inherited susceptibility and/or physiological dysregulation in all three systems, particularly for catechol-O-methyl transferase (COMT) genes, the glucocorticoid and the related mineralocorticoid receptors (NR3C1, NR3C2), and the purinergic 2X4 (P2X4) ion channel involved as a sensory receptor for muscle pain and fatigue and also in upregulation of spinal microglia in chronic pain models. Methodological concerns for future research, including potential influences of comorbid clinical depression and antidepressants and other medications, on gene expression are also addressed.

## 1. Introduction

The concept that fibromyalgia syndrome (FMS) may involve inherited susceptibility is not new, nor is the related hypothesis that FMS pathogenesis involves a genetic susceptibility combined with environmental exposure that triggers further changes in expression of the same gene(s) or other interacting genes [1–3]. These environmental events might include one or several of the following: traumatic injury, bacterial or viral infection, surgery, or chronic intermittent life stressors. All of these environmental events increase stress exposure (defined as the external events themselves) and distress (defined by the individual's physiological and emotional responses to stressful events). Increased distress is also a consequence of the chronic pain of FMS itself and its cost to normal work, family, and social functioning [4, 5]. This distress may be greater in those individuals with specific biological predispositions that alter function of the two main stress pathways, the sympathetic (adrenergic) and hypothalamic-pituitary-adrenal (HPA) axes [6]. Distress is certainly greater in those with psychobiological predispositions, such as depression, anxiety, and pain catastrophizing, all of which are intercorrelated with each other and with severity of disability [7]. The focus of this report is on the genetic factors that may underlie these susceptibilities (inherited DNA) and on the gene-environment interactions that can lead to altered gene expression (mRNA) and thereby change the neural and immune pathways that regulate the primary symptoms of muscle pain and fatigue.

In order to rigorously test for possible genetic and gene expression contributions in FMS, it is vital that within the broadly inclusive FMS patient definition, key patient subgroups must be carefully defined to be as homogeneous as possible. Diagnosis of FMS is currently undergoing some evolution, but is based largely on subjective reports of widespread pain involving muscles and joints that last for 3 months or longer. Research on genes and gene expression in FMS may identify objective biomarkers for the disorder, as well as indicate pathways that are dysregulated and thus are potential targets for therapeutic intervention. Many patients meeting the older American College of Rheumatology (ACR) criteria for FMS, which requires widespread hyperalgesic response to Tender Point testing [8], also meet the Fukuda et al. and Reeves et al. [9] criteria for chronic fatigue syndrome (CFS); this percentage has been reported from 21–80%, with less overlap in primary care and population studies but approaching 70% in specialized referral clinics [10]. It has even been suggested that FMS, CFS, and other overlapping somatic disorders like irritable bowel syndrome (IBS), and other noncardiac chronic pain conditions should be classified as a single disorder labeled “bodily distress syndrome” [11]. The more recent recommendations for clinically defining FMS without requiring Tender Point testing and highlighting fatigue as one of the central constellation of symptoms [8, 12, 13] is likely to further increase the overlap of CFS and FMS. With the broader definition of FMS, however, there is also a greater likelihood of inclusion of subgroups with differing etiologies involving disparate genetic and gene expression profiles. Because of the overlap in these syndromes, in the present report, we will summarize the literature on genetic allelic differences and gene expression in both FMS and CFS, with an emphasis on stress-related genes that may indicate dysregulation in three interacting neural pathways: the adrenergic nervous system known to be activated during many types of physical and mental stress, the serotonin and HPA axis linked to distress through depression, and the purinergic and other ion channel receptors (e.g., acid sensing ion channel (ASIC) and transient vanilloid-1 or capsaicin receptors (TRPV1)) that our own research linked to increased pain and fatigue symptoms in patients with CFS and comorbid FMS following moderate physical activity.  Table 1 lists the various stress-related genes addressed in this review.

## 2. Functional Importance of Adrenergic and Ion Channel Receptors in the Pain and Fatigue of FMS and CFS

Adrenergic receptors are best known for their function as components of the efferent sympathetic pathway, with greatest emphasis on the heart and vasculature as the target organs. A change in central sympathetic activity initiates an increase or decrease in neural activity to *β*-1 receptors in the myocardium altering heart rate and contractile force, and to *α* receptors in arterial and venous vessels, influencing constriction and dilation and thereby altering blood flow to specific tissues and venous return to the heart. Equally important in the regulation of blood flow are the vascular *β*-2 receptors which enhance dilation when activated, a response that principally occurs in response to increased circulating levels of norepinephrine and epinephrine. The levels of these circulating amines reflect the sum of both adrenal production and local release from postganglionic sympathetic nerve fibers, but may also reflect the rate of metabolism which varies as a function of key enzymes, particularly catechol-O-methyl transferase (COMT). What is less commonly noted is that vascular *β*-2 receptors are also critical components of the metabolic pathway that causes blood flow to increase in a specific muscle when that muscle's activity increases and more metabolites of activity are produced (local autoregulation). Because this local autoregulation is so firmly tied to increases and decreases in local metabolic activity, this necessarily involves communication between the vascular *β*-2 receptors and the sensory ion channel receptors that are involved in detection and signaling of increases in metabolites of muscle activity that occur as we exercise first to fatigue and then (if exercise is unabated) to muscle pain.

In addition to their role in efferent activity, both *α*-2 and *β*-2 adrenergic receptors have been implicated in animal models of neuropathic and inflammation-induced pain [14–17]. Rahman et al. [18] conclude that in healthy individuals, a spinal pathway including *α*-2 receptor activity provides a tonic inhibition of neuronal responses to mechanical pain, and this inhibition is lost after peripheral nerve injury. In regard to *β*-2 adrenergic receptor involvement, this may differ depending upon whether the chronic pain state is new or well established. Coadministration into the temporomandibular joint (TMJ) of the inflammatory agent, carrageenan, together with a *β*-2 adrenergic receptor antagonist, prevents the chronic TMJ hyperalgesia that typically follows carrageenan alone [17]. However, in established neuropathy induced by sciatic nerve cuff, chronic administration of *β*-adrenergic receptor agonists reduce mechanical allodynia via *β*-2 receptor activity [19].

Until recently, no solid candidates for the molecular pathways signaling these sensations that we all have experienced have been confirmed. A significant breakthrough occurred, however, when McCleskey and colleagues found that ASIC3 receptors have the appropriate characteristics to detect such ischemia; they are extraordinarily sensitive to protons (acid), and they are expressed highly in dorsal root ganglion (DRG) neurons that innervate skeletal muscle and the myocardium [20, 21]. They noted that ASIC3 sensitivity to acid is greatly enhanced if extracellular lactate and adenosine triphosphate (ATP) are also present. Subsequently, Sluka and colleagues confirmed that ASIC3 is also important in detecting hyperalgesia in skeletal muscle using a model of chronic pain induced by inflammation. They also demonstrated that exercising their mice to fatigue made these animals more likely to develop hyperalgesia after acidic saline injections [22–24]. In related work, Hayes, Kaufman and colleagues confirmed in cat that ATP-sensitive P2X and ASIC but not P2Y receptors on muscle afferents are important in pressor responses and changes in muscle blood flow induced by chemoreflexes similar to those occurring during muscle activity [25, 26]. Extending this basic research on ASIC and P2X receptors, Light and colleagues [27] recently showed that mouse DRG neurons innervating muscle respond poorly to a single metabolite like lactate, but respond much better to physiological levels of *combinations* of the normal metabolites of muscle work including acid, ATP, and lactate. By applying specific antagonists for ASIC3, P2X, and TRPV1 receptors, Light et al. also confirmed that these different ion channel receptors *work together in concert* to signal increases in the levels of these metabolites. Furthermore, two types of DRG neurons innervating muscle were seen: one that responded to low metabolite levels (and may signal fatigue), and another that responded maximally to higher metabolite levels (and thus may signal muscle pain). 

For the P2X receptors, in addition to their role as part of a receptor complex detecting potentially painful levels of metabolites, they also contribute to the establishment of chronic pain state by influencing the function of spinal microglia [28]. Microglia become activated under many conditions, including trauma, inflammation, and infection, during which they release chemical mediators including proinflammatory cytokines. Following experimentally induced inflammation, peripheral nerve injury or autoimmune models of neuritis, P2X4 expression is enhanced in activated spinal microglia, and administration of P2X inhibitors leads to a reduction in the hyperalgesia that otherwise follows such injury [29–32]. This research on P2X4 involvement in chronic pain conditions has been somewhat limited by the lack of a specific P2X4 receptor antagonist and has thus turned instead to mouse models where the P2X4 gene has been disrupted. These P2X4 −/− mice show normal responses to acute noxious stimuli, but reduced responses to both chronic inflammatory pain induced by intraplantar injection of Freund's adjuvant and to neuropathic pain induced by spinal nerve injury [29, 32]. Furthermore, following inflammatory treatments, P2X4-deficient mice fail to show the expected increase in prostaglandin E2, a potentially important step in the initiation of inflammatory pain [31]. 

As noted by Light et al. [27], a third ion channel receptor is part of the metabolite detecting complex, TRPV1, or the capsaicin receptor. Fujii et al. [33] have reported that inflammatory pain and delayed onset muscle soreness in rats is blocked by both ASIC and TRP receptor antagonists. Thus, upregulation of P2X4, ASIC3 and TRP receptors in the DRG could contribute to exaggerated sensitivity to metabolites linked to fatigue and muscle pain sensations, and upregulation of the same receptors on microglia could play an important role in the establishment of chronic pain following inflammation, nerve injury or autoimmune conditions. 

## 3. Genetic Polymorphisms Linked to FMS Susceptibility or Subgroup Differences

Early research on genetic susceptibility in FMS focused on serotonergic pathways. The rationale for this focus was partly that effective and approved pharmacological treatments for FMS included drugs commonly classified as serotonin and norepinephrine reuptake inhibitors (SNRIs). Although a few of the studies have been positive [34], the majority of investigations on genes involving serotonin function have not shown any association with FMS. For example, Frank et al. [35] found no evidence of any difference in frequency of polymorphisms involving the serotonin receptors 3A or 3B while Tander et al. [36] similarly saw no FMS difference in serotonin receptor 2A polymorphisms, and Gürsoy [37] found no links to serotonin transporter gene variants. In the most recent investigation, reported by Nicholl et al. [38] and Holliday et al. [39], two serotonergic gene SNPs for serotonin receptor 2A and serotonin synthesis gene TPH2 were related to somatic symptoms in the general population and in patients with chronic wide spread pain (CWP). There were of course very different clinical definitions used for FMS in the negative studies versus CWP and somatic symptoms in the latest positive studies, and none of these studies have clearly defined the characteristics of the subgroups where the serotonin SNPs are seen. It was recently shown that female patients with CFS only have increased serotonergic tone (defined by increased prolactin response to tryptophan infusion) while those with CFS who have comorbid FMS (CFS+FMS) have normal serotonergic tone by this test [40]; note that this study used the older ACR criteria for FMS including Tender Point exam. Thus, any future research on serotonergic genes or other serotonin-related biomarkers in FMS using the new broader definition for the disorder must acknowledge the importance of subgrouping based on either Tender Points or some other tests of hyperalgesic response.

The second system to receive intense attention for genetic polymorphisms was the other system directly altered by SNRIs, the adrenergic nervous system. Here too outcomes have generated mixed positive and negative findings [41], which we believe are largely due to differences in FMS samples, possibly involving whether the sample included larger versus smaller percentages who had comorbid CFS. The adrenergic gene that has received the most attention in FMS to date is the val(158)met single nucleotide polymorphism (SNP) for COMT, the primary enzyme that metabolizes and inactivates catecholamines. Gürsoy et al. [42] reported that specific polymorphisms for this COMT gene differed in frequency among FMS patients versus controls. They further reported that these polymorphisms were unrelated to clinical depression or other psychiatric disorders in their sample. Cohen et al. [43] replicated this finding in a larger sample of 209 female FMS patients compared to 152 of their own nonaffected relatives, and they showed that the met allele was linked to increased number of positive Tender Points. More recently, Finan et al. [44] have linked specific COMT haplotypes to catastrophizing, suggesting that this gene influences cognitive coping strategies. Other studies have shown that these as well as other gene haplotypes associated with reduced COMT enzymatic activity are linked to greater sensitivity to experimental pain among healthy women, and greater risk of developing musculoskeletal disorders including FMS and temporomandibular disorder (TMD) [45–48]. In contrast, studies using patients having the broader diagnosis of CWP with no attention to Tender Point have generally not found any association with COMT haplotypes even with very large samples [49, 50]. This appears to suggest that the characteristics of hyperalgesia and/or allodynia as reflected by responses to Tender Point testing and to experimental pain tasks may be importantly related to the COMT haplotypes and their links to development of TMD and FMS. Further large-scale prospective longitudinal research on the role of COMT-related genes in the pathogenesis of TMD and FMS is in progress, and will provide more definitive tests of the role of these genes.

Additional research on adrenergic genes has indicated that haplotypes influencing *α*-1a adrenergic receptor and the *β*-2 adrenergic receptor differ in patients with FMS. Vargas-Alarcón et al. [51] examined SNPs for *α*-1, *β*-2, and *β*-3 receptors in samples of Spanish and Mexican FMS patients versus controls. Of these, only the *β*-2 adrenergic receptor SNPs were differentially expressed in the FMS patients from both locations while in the Spanish patients only, there was also increased frequency of one of the 3 *α*-1 adrenergic receptor SNPs studied. In a prospective study, Herlyn et al. [52] reported an association of the same *α*-1 receptor polymorphism, the rs1048101, with increased risk of development of complex regional pain syndrome following fracture of the radius. Polymorphisms for the adrenergic receptors *α*-2A and *α*-2C have both been linked to IBS and to broader somatic complaints including pain in these patients [53]. Xiao et al. [54] found that the Gly16Arg polymorphism for the *β*-2 adrenergic receptor was less frequent in FMS than controls. Those FM patients carrying the alternative Arg16Arg polymorphism showed lower intracellular cyclic AMP functioning. It is important to note that Nackley et al. [47] found that *β*-2 adrenergic receptors are involved in the increase in pain sensitivity that results from diminished COMT activity. Thus, it is possible that the combined presence of genes that downregulate both *β*-2 receptors and COMT activity in the same individual may more greatly enhance pain sensitivity and risk for FMS than either gene polymorphism individually. Our research group [55] demonstrated short-term reduction in clinical pain ratings in patients with FMS (including 40% with comorbid CFS) and TMD following *β*-receptor blockade with low-dose propranolol (which blocks both *β*-1 and *β*-2 receptors). Subsequently, Tchivileva et al. [56] used a double-blind crossover design to compare one week of propranolol versus placebo treatment, and similarly found that total pain ratings of TMD patients were decreased by the *β*-adrenergic antagonist; they also found that propranolol did not alter sensitivity to experimental pain in all TMD patients, but did reduce it in those with the COMT haplotype linked to decreased enzyme production.

Altogether, these findings indicate that genetic haplotypes for *α* and *β*-adrenergic receptors and COMT confer an inherent susceptibility and are related to risk of developing chronic musculoskeletal and gastrointestinal pain disorders including FMS, TMD, and IBS. Future FMS research should include larger and better characterized samples so that it can be determined whether these risks differ depending on the presence or absence of comorbid CFS, IBS, orthostatic intolerance, and other clinical factors linked to altered autonomic/adrenergic function.

Another central pathway that has received some attention among genetic studies in FMS and related disorders is the dopaminergic pathway. Zubieta et al. [57] reported that the SNPs linked to reduced COMT activity were associated with changes in effects of mu-opioid transmitters on experimental pain. Wood et al. [58] had observed that FMS patients show abnormal dopamine responses to pain. Although Triester et al. [59] found that healthy subjects with specific SNPs for the dopamine transporter gene were less tolerant to noxious cold, Ablin et al. [60] found no evidence for differences among Israeli FMS patients in haplotypes for the dopaminergic transporter gene or for the substance P receptor. However, genetic variants of the dopamine receptor-4 (DRD4) have been associated with FMS or with an overlapping disorder, migraine headaches, in several studies [61–63]. Other dopamine receptor and transporter gene SNPs were not linked to migraine in Spanish samples [64, 65] but dopamine transporter and dopamine beta-hydroxylase gene SNPs did show an association to migraine with aura in other European samples [66].

The purinergic receptors are another broadly influential pathway deserving of attention in genetic studies of FMS and other disorders involving chronic pain and fatigue. IFN-gamma has been shown to upregulate P2X4 receptors (both expression and protein) in vascular endothelial cells [67]. Furthermore, flow-mediated dilation is impaired in P2X4 knockout mice, which have higher BP and have altered nitric oxide (NO) function, even excreting less NO in urine [68]. In one study, a polymorphism linked to lower expression of the P2X7 receptor was associated with subgroups of patients, with both systemic lupus erythematosus and rheumatoid arthritis having poor apoptotic function [69]. Also, SNPs for the P2X7 receptor gene have been associated with clinical anxiety and both monopolar and bipolar depression disorders [70–73]. Yet to date, there have been no published studies of which we are aware examining genetic variants of any purinergic receptor genes in FMS, CWP, CFS, IBS, migraine, or any other overlapping multisymptom disorder.

## 4. Gene Expression Research

Gene expression as opposed to genetic SNPs (mRNA rather than DNA) is typically determined through one of two major methods: microarray where the full genome is assessed together, and quantitative real-time polymerase chain reaction (qPCR) where selected primers for specific genes are examined individually. The micro-array approach is attractive for multisymptom disorders like FMS where dysregulation in many physiological systems may potentially be involved, simply because all of them can be examined at once. The primary drawback to microarray for FMS research, however, is that due to examining such a huge number of outcome measures in the same study, it is necessary to use a very large patient and control sample and/or to lump together multiple genes to assess as a single pathway, in order to control for a tremendous statistical problem of false-positive findings. Some authors ignore the false positive problem, and report each gene as though it were an independent test; for example, Gow et al. [74] studied only 8 CFS patients versus 7 controls using the full 33,000 gene sequences from the Affymetrix array, and report that these groups differ significantly in 366 genes (roughly 1% of genes tested). This is very likely one of the factors that has led to frequent nonreplication of results. In contrast, the number of genes examined using qPCR is usually 1–40, and they are typically selected on the basis of representing a specific pathway where prior research has indicated a functional difference. Several studies have reported that from 1–6 genes related to immune function were upregulated in leukocytes of CFS patients compared to controls [75–78]. One option, employed by Kerr and colleagues to examine patients with CFS, is to use microarray for an initial study to identify pathways where multiple gene show differential expression, and then follow this with qPCR on the strongest candidate genes within those pathways [79, 80]. However, Frampton and colleagues [81] have recently found that even using a 44-gene profile generated from their original research, its utility in prospectively distinguishing CFS patients from controls in a new test was only fair (correctly identifying only about 60% of those tested).

One important advantage for gene expression protocols is that one can examine the mRNA levels before and after a challenge. Many genes are upregulated or downregulated quite rapidly in response to normal physiological events, like physical exercise, pain, emotional stress, exposure to an infectious agent, toxins, and many others. In this way, the dysregulation that may be too subtle for detection in the resting state can be revealed. In an early investigation using micro-array to examine gene expression changes in response to an exercise challenge, the Centers for Disease Control (CDC) research group elected to use a moderate bicycle exercise task (using 70% percent of age-predicted maximum heart rate) rather than a maximal exercise task, which has long been the preferred option for exercise scientists focusing on the cardiovascular system [82]. Their rationale for this decision, which is compelling, was that a submaximal exercise task has 3 key advantages over a maximal exercise test. First, even debilitated patients can complete moderate exercise on a stationary bicycle. Second, it can be performed for an extended period of time (here, 20 min) and duration can be matched across subjects while maximal tests are briefer (7–12 min) and the duration for deconditioned CFS patients may average only 60–75% of that in controls, which could by itself contribute to gene expression differences. Third, this type of exercise is more typical of the activities of daily living that cause postexertional malaise (defined as worsening fatigue, pain, or feelings of sickness), a key symptom of CFS. This CDC-based study observed increased expression of genes in a number of pathways at 24 hours after the exercise in the 5 CFS versus 5 controls, of which the most relevant were ion transport and ion channel activity genes. A more recent case-control study with another small sample (8 CFS patients versus 7 controls) using the same exercise protocol but with qPCR assays for mRNA at 6 hours after the exercise showed increased activity in a pathway linked to complement activation [83]. Using a different Bayesian approach to analyze the same data set, which looked for genes that differed at baseline as well as in response to the challenge, Lin and Hsu [84, 85] found differences in the glucocorticoid receptor gene NR3C1. It should be noted that both Goertzel et al. [85] and Rajeevan et al. [86] had previously reported that NR3C1 haplotypes were differentially present in CFS patients while Macedo et al. [87] observed that a related gene SNP for NR3C2, the mineralocorticoid receptor, was differentially present in FMS patients and this was linked to lower baseline gene expression of both NR3C1 and NR3C2.

**Figure 1:**
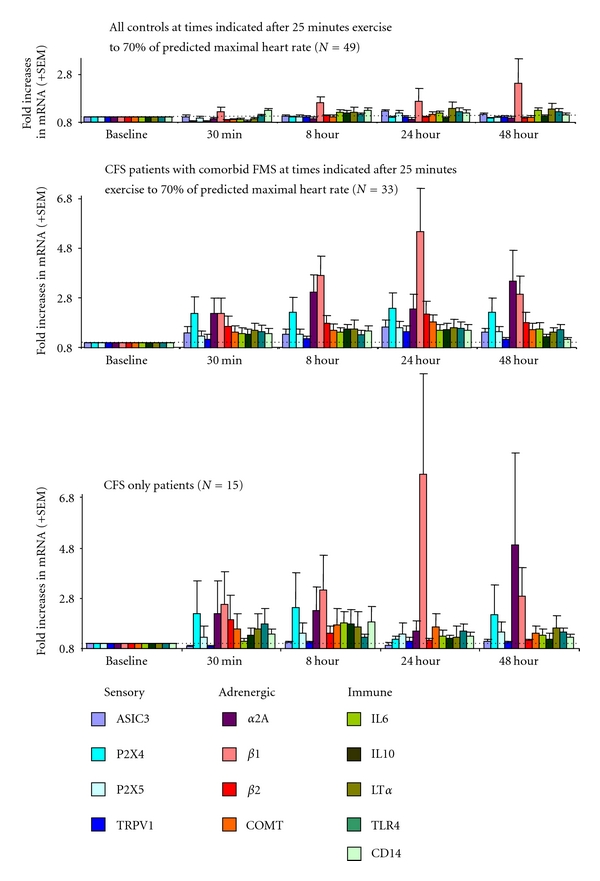
Increases in leukocyte gene expression at 30 minutes, 8 hours, 24 hours, and 48 hours after moderate intensity exercise in healthy controls (top), CFS+FMS patients (middle), and CFS-only patients (bottom). Data for each gene (see color codes) are depicted as fold increases from preexercise baseline at each of the 4 sampling times; thus, 2.0 = twice as much expression as at baseline, and so forth. CFS+FMS and CFS only significantly greater than controls for P2X4, P2X5, TRPV1, *α*-2A, *β*-1, *β*-2 adrenergic receptors, COMT, and IL10 assessed as area under the curve across all 4 postexercise sampling times (*P* < 0.05). CFS+FMS greater than CFS-only and controls for ASIC3 (*P* < 0.05). Data are adapted from results reported by Light et al. [89].

To date, the only research examining leukocyte gene expression responses after exercise in patients with FMS-only and patients with CFS plus comorbid FMS has been by our University of Utah research team [88–90]. The 1994 Fukuda et al. criteria plus the Reeves criteria [9] were used to define CFS, including persistent or relapsing fatigue of 6 months duration or longer that results in substantial restriction of life activities, and is accompanied by at least 4 of these 8 additional symptoms: unusual worsening of fatigue, pain, or general unwellness following exertion, impaired memory or concentration, muscle pain, joint pain, unrefreshing sleep, change in headaches, sore throat, and tender lymph nodes. To define FMS, patients had to have widespread muscle/connective tissue pain, including all 4 body quadrants (bilateral, upper, and lower body) for 3 months or longer, and hyperalgesic responses to pressure at Tender Points (with pain reported for at least 11 of 18 Tender Points) [91]; activity-restricting fatigue was not involved in the definition of FMS, and the FMS-only group reported only milder fatigue that rarely limited life activities. We modeled our 25-minute moderate exercise stressor after the one used by the CDC research, but extended the blood sampling times to include 0.5, 8, 24, and 48 hours after the exercise, corresponding with the typical duration of after the exertional malaise reported by many CFS patients. In our recent investigation, [89], we have also employed a much larger sample including 48 patients with CFS only or CFS with comorbid FMS, and 18 patients with FMS only who report some daily mental or physical fatigue but do not meet criteria for CFS. As our primary comparison group, we tested 49 healthy controls who were age-matched to the CFS and CFS+FMS patients. Because of the risks and problems associated with asking patients to be withdrawn from antidepressant medications, 30 of 48 CFS patients (63%) and 13 of the 18 FM-only patients (72%) were tested while continuing their usual antidepressants; however, to mitigate possible confounding effects of these medications, 11 of 49 control subjects (23%) also had been diagnosed and were currently on medication for clinical depression. We also included another deconditioned patient group, 20 multiple sclerosis (MS) patients who reported daily fatigue that significantly impacted functioning [90]. The genes selected for qPCR assay included adrenergic receptors and COMT, sensory ion channel receptors P2X4, P2X5, ASIC3, and TRPV1, and several cytokine and immune genes including IL10, IL6, lymphotoxin-*α* (LTa), Toll-like receptor-4 (TLR4), and cluster of differentiation-14 (CD14). At baseline, there were no differences in gene expression between any of the CFS groups or the MS group versus the controls. As depicted in Figure 1, healthy controls (including those on medication for clinical depression) showed no significant increases after moderate exercise in any of the genes under study, despite a trend based on some healthy subjects showing an increase in *β*-1 adrenergic receptor expression. In contrast, the majority of patients with CFS only or CFS+FMS showed postexercise increases in most of the sensory ion channel genes and all of the adrenergic receptor and COMT genes, as well as IL10, differing from controls in all of these responses. Importantly, patients with CFS only and CFS+FMS showed nearly identical increases in all genes except ASIC3, which increased only in the CFS+FMS group. These postexercise increases were evident as rapidly as half an hour after the moderate exercise task, and persisted for the full 48 hours.

These differences from controls were also evident when comparisons involved reduced samples matched for actual work performed to attain 70% of their predicted maximum heart rate, thereby controlling for differences in fitness level [89]. Postexercise severity of clinical pain and fatigue ratings in CFS-only and CFS+FMS patients were correlated with increases in gene expression, especially with P2X4, *α*-2A and *β*-2 receptors (*r  *=  +0.43, +0.48, and +0.44 for pain, and *r  *=  +0.51, +0.60, and +0.47 for fatigue, resp.; *P  *<  0.01) [89].

**Figure 2:**
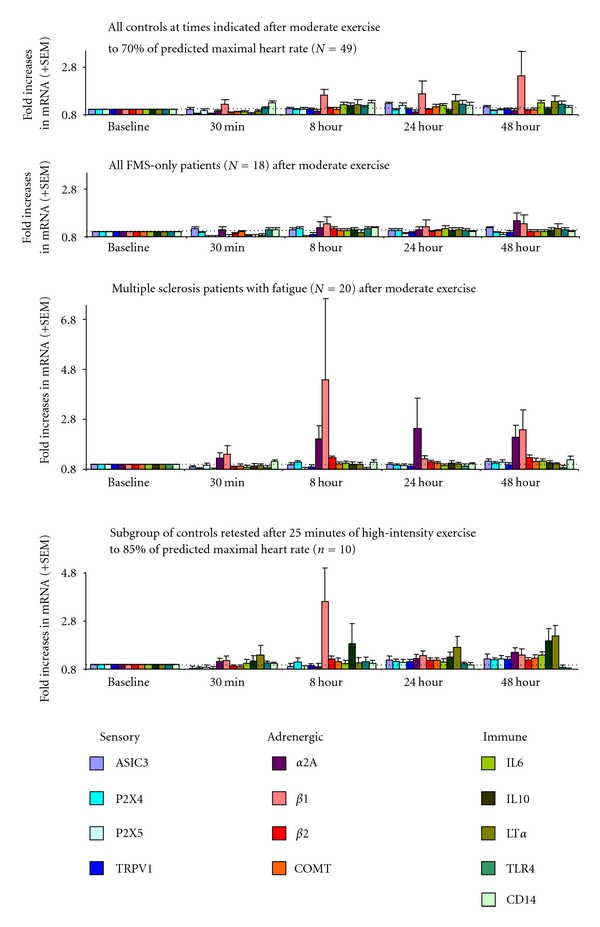
Increases in leukocyte gene expression at 30 minutes, 8 hours, 24 hours, and 48 hours after moderate intensity exercise in healthy controls (top), FMS only patients (upper middle), multiple sclerosis patients (lower middle), and after higher intensity exercise in healthy controls (bottom). FMS-only do not differ from moderate exercise controls in any gene. MS patients significantly greater than moderate exercise controls for *α*-2A, *β*-1 and *β*-2 adrenergic receptors (*P* < 0.05). High-intensity controls significantly greater than moderate intensity controls for adrenergic receptors, LT*α* and IL10 (*P *< 0.05) but not for any sensory gene. CFS+FMS combined with CFS-only patients from Figure 1 significantly greater than high intensity controls for ASIC3, P2X4, TRPV1, *α*-2A and *β*-2 adrenergic receptors, and COMT (*P* < 0.05). Data are adapted from Light et al. [89], White et al. [90], and unpublished observations.

Unlike those with CFS+FMS, patients with FMS only showed no significant increases in any gene after moderate exercise (see Figure 2). The other patient group, the MS patients, showed modest and transitory increases in *α*-2A and *β*-1 adrenergic receptors, but these were significantly lower overall than the increases in the CFS groups, and they had no increases in any of the sensory ion channel genes. The MS group responses were in fact very similar to 10 healthy controls who exercised for 25 minutes at much higher intensity, 85% of their age-predicted maximum heart rate, who likewise increased *β*-1 adrenergic receptor expression, but not any sensory receptor. The very modest responses of this high-intensity control group contradict the possibility that, although our moderate exercise task was individually adjusted to evoke a similar cardiovascular response, it was still a more intense stressor for the CFS patients and this resulted in their greater gene expression changes. 

Increases in P2X4, P2X5, TRPV1, and ASIC3 plus exaggerated COMT, *α*-2A, and *β*-2 adrenergic receptor expression following moderate exercise have been a unique profile in CFS and CFS+FMS patients, not evident in any other patient group with chronic fatigue or pain that we have examined to date. Both the adrenergic receptor and ion channel gene expression was directly correlated with the severity of postexertional fatigue and pain reported by the CFS group. It is notable that our gene expression responses did identify a subgroup among the CFS patients showing decreases rather than increases in *α*-2A adrenergic receptors lasting for 48 hours; this subgroup did not show increases in any other genes, and they were much more likely to have a clinical history of orthostatic intolerance than other CFS patients. These clinical and gene expression differences provide a helpful starting point for individualized treatment options. Surprisingly, despite their shared fibromyalgia and the fact that both groups reported postexercise increases in pain and fatigue, the FMS-only group was unlike the CFS+FMS group and showed no postexercise increases in these genes.

However, the FMS-only patients did show evidence of dysregulation in the sensory ion channel pathway at baseline prior to exercise. These patients showed increased expression of both P2X4 and TRPV1 receptors at baseline, along with increased IL10 [89]. It is possible that these patients differed from the CFS+FMS primarily in their ability to cope with their sensations of fatigue. If so, they would report less fatigue severity as a chronic symptom, and be able to remain more active in daily life activities. Even though all patients and controls were instructed to refrain from formal exercise beyond slow walking for 48 hour before and 48 hours after our exercise test, the FMS-only patients may have been functional enough to have baseline responses that were nevertheless elevated by more activity while the CFS+FMS and CFS-only groups did not. This possibility might be best addressed in future research by use of activity logs and objective documentation such as with the Actigraph monitoring systems.

We have interpreted these observations as indications of dysregulation in the primary metabolite-detecting neural pathway that senses products of muscle activity including protons, lactate, and ATP. Large-scale increases in P2X4, P2X5, TRPV1, and ASIC3 receptors in the DRG would potentially increase sensitivity to even low levels of these metabolites such that even activity as mild as upright posture and slow walking in CFS or CFS+FMS patients could produce sensations of fatigue and muscle pain that normal individuals would only feel during extreme activity [92]. One major problem with this research is that directional changes in gene expression are tissue specific, and it is not possible to obtain samples of DRG or other neural tissues from living human individuals without causing irreversible damage. Leukocytes, in contrast, are easily obtained without risk on a repeated basis, but we are compelled to use them as indirect markers of these critical neural cells. To reinforce our interpretation, we must depend on research in animal models where the hypothesized parallel changes in leukocyte and DRG genes can be directly confirmed or refuted. A recently emerging literature, however, supports the use of gene expression profiles from leukocytes as useful surrogates to neuronal expression for genes linked to pain affect and mood [93–95], thus strengthening use of this approach in the context of FMS. In all studies of gene expression, there is also the additional limitation that this is an indication that processes have been engaged in an effort to increase or decrease the production of a specific receptor or other protein, and that changes in actual protein levels may not always occur.

## 5. Methodological Issues for Future Research on Genetics and Gene Expression of FMS

One of the difficult issues for this area of research is that many patients with FMS, CFS, and related multisymptom disorders develop clinical depression secondary to their chronic disabling pain and fatigue symptoms, which make performing the usual activities of work and family life difficult or even impossible. When attempting to separate biological effects of depression from the effects of their primary disorder, some investigations have elected to study only those FMS patients who do not meet criteria for clinical depression [40, 55]. This strategy is vulnerable to the criticism that by eliminating FMS patients with depression, the sample may be biased and nonrepresentative, and especially may include only less severely affected FMS patients. Another approach which may be more expensive in terms of resources but more valid is to include patients with clinical depression who do not meet criteria for FMS, CFS, or any other multisymptom disorder as a second comparison group, along with healthy controls. This latter approach could be especially useful when attempting to match the patient and comparison groups for a number of other characteristics, including current or prior use of certain medications, affective state, and use versus avoidance of traditional medical treatment for chronic conditions. Using patients with clinical depression who do not meet criteria for FMS as a comparison group is also extremely important because of the hypothesis that CFS and FMS may be severe somatization variants of major depressive disorder, a possibility which many CFS and FMS patients dispute [96, 97]. A different concern applies to investigations in which the FMS patients are required to stop all their medications for an extended period of time, because the more severely affected patients may be unwilling or unable to do this. However, if all FMS patients are tested while using a specific medication and none of the controls are tested on this medication, gene expression differences between groups could be due to the medication and not the diagnosis. For example, Salemi et al. [98] found that both kappa- and delta-opioid receptor genes had higher expression in skin of patients with FMS versus healthy controls. However, because the report does not clarify medication use or recent cessation, the differences could also be due to more FMS patients either currently using opioid medications or, equally problematic, having receptors that are in a “withdrawal state” due to stopping these medications for only a few days prior to the study. In contrast, one of the better designed studies to date by Macedo et al. [87] reported reduced leukocyte gene expression of both the glucocorticoid receptor NR3C1 and the mineralocorticoid receptor NR3C2 in patients with FMS, all of whom stopped their antidepressants for at least 2 weeks prior to testing. This study was also especially well designed in that they concurrently tested the functionality of the corticosteroid pathway by measuring basal serum and saliva cortisol levels and examining responses to dexamethasone. Finally, they looked for genetic polymorphisms for these receptors, and found an excess of one allele for the mineralocorticoid receptor, which was then linked to diminished expression of both these related genes NR3C2 and NR3C1. In our research on gene expression differences in patients with FMS (including 72% tested on SSRI or SNRI antidepressants), we have recently compared this group to patients with moderate to severe clinical depression persisting despite SSRI and SNRI treatment as well as to healthy individuals including a subgroup with milder depression that was well controlled by antidepressants (Light et al., unpublished observations). We observed that the FMS and the treatment-refractory depressed patients differed from the controls in expression of a number of the same genes, including similar increased expression of the ASIC1a gene. In mice, genetically disrupting ASIC1a or administering an ASIC1a antagonist reduced depressive-like behaviors in the forced swim test while restoring ASIC1a to the amygdala with a viral vector reversed this effect [99]. In our FMS and depressed patients, this similar increase in ASIC1a expression may reflect common neural dysregulation that contributes to both of these disorders, or instead may reflect the consequences of persisting depressive symptoms. Importantly, the effects do not appear linked to antidepressant use, since this factor was not reliably related to ASIC1a expression in any of our statistical models. 

One weakness of the exemplary study by Macedo et al. [87] was a failure to examine their patients for clinical information that might more closely identify the specific clinical characteristics of the FMS who showed this genetic and genomic profile; specifically, it would have been very desirable to know whether they also met criteria for comorbid CFS or other overlapping disorders. We feel very strongly that in a heterogeneous syndromic disorder like FMS, research is greatly strengthened when meaningful subgroups can be differentiated. The findings can then be more specifically useful for clarifying factors linked to pathogenesis and prognosis. The well-defined subgroups together with the genetic and genomic data, when combined, can also provide a clear rationale for individualized physiological targets for treatment. 

To date, most of the research summarized above has taken a single blood sample from patients and controls, and examined gene SNPS (DNA) or gene expression (mRNA). Gene expression responses to exercise have rarely been employed, and gene expression responses have not been assessed to any other types of challenge. Future research should continue to examine gene expression responses to exercise and other physical challenges, including pain stimuli and psychological stressors which have particular relevance in hypotheses about FMS onset and progression. When exercise is used as the challenge, it is important to consider both submaximal and maximal exercise tasks as valid options, depending upon the specific goals of the study. We reiterate that submaximal exercise is closer to the usual physical activity of daily life that leads to worsening of muscle pain and fatigue for 24–48 hours in patients but not healthy individuals, and thus may be better than maximal exercise at revealing physiological pathways that are dysregulated in patients versus controls. If possible, longitudinal research should be encouraged in which both genetic and gene expression measures are obtained in subjects who are still healthy but have genetic SNPs previously linked to FMS susceptibility (for NR3C2, serotonin, COMT, or other haplotypes), and gene expression responses reexamined after a period of years when a subgroup of these individuals may meet criteria for FMS.

As a final point of emphasis, there is a critical need to continue to integrate genetic and genomic clinical research with closely associated research in animal models of FMS—what has been called “reverse translational research.” At this time, for example, research with animal models is critical in the development and testing of new receptor antagonists, such as a specific antagonist for P2X4 receptors. This type of pharmacological tool could be important both for its research and therapeutic possibilities in FMS. Genetic mutant and knockout mouse models are also extremely valuable to help elucidate multiple physiological pathways that are influenced by any genetic or genomic variations of interest. As stated previously, virtually all gene expression research in humans is based on leukocytes, and must acknowledge the limitation that this may or may not parallel gene expression changes in other organs and tissues, like the DRG, spinal cord, or specific brain regions of interest. Parallel studies in animal models can explore gene expression changes in neural tissues and thus greatly reinforce the clinical findings. Thus, much more translational clinical research and reverse translational basic research on adrenergic and purinergic genes and gene expression is encouraged for expanding our understanding of the physiological and environmental factors that initiate and maintain the chronic symptoms of FMS, and to lay the foundation for treatments that are more effective and have fewer side effects for each individual patient.

## Figures and Tables

**Table 1:** Stress-related genes studied for DNA or mRNA effects in FMS, CFS, or related disorders.

Adrenergic receptors		*α*-1, *α*-2A, *α*-2C, *β*-1, *β*-2, *β*-3
Catechol-O-methyltransferase		COMT
Cytokines		IL6, IL10, LTa, CD14, TLR4
Dopamine receptors, enzyme, and transporter		DRD1, DRD2, DRD3, DRD4, DBH, SLC6A3
Glucocorticoid and mineralocorticoid receptors		NR3C1, NR3C2
Ion channels	Purinergic	P2X4, P2X5, P2X7
	Acid sensing	ASIC1a, ASIC3
	Transient vanilloid receptor	TRPV1
Opioid receptors (kappa and delta)		OPRK1, OPRD1
Serotonin receptors, synthesis gene and transporter		HTR2a, HTR3a, HTR3b, TPH2, SLC6A4
